# Dr. Andrew J. Martin

**Published:** 1916-01

**Authors:** 


### OBITUARY

**Readers are requested to report promptly the death of all physicians in Western New York, or former residents of this region, or graduates of any medical school in Western New York, and to notify the families of the deceased of our desire to publish adequate Obituary notices.**

**Dr. Andrew J. Martin,** Buffalo 1887, died at the German Deaconess's Hospital in Buffalo, following an operation, November 24, aged 55. He was born in the town of Clarence, Erie Co., N. Y., and was graduated at the Buffalo State Normal School. He taught school for several years before studying medicine. After graduating in medicine, he practiced for five years in Clarence and then studied at Heidelberg for two years and served in the Vienna General Hospital for a year, returning to America in 1897 and locating in N. Tonawanda, where he has practiced ever since. He was a member of the State and County societies, of the Twin City Academy of Medicine and of the local chapters of the Masons and Odd Fellows. He was coroner of Niagara Co. for three years, Helath Officer of N. Tonawanda for three terms and for many years served on the local Board of Health. His loss is much regretted.

